# Passive enhanced safety surveillance for Vaxigrip and Intanza 15 µg in the United Kingdom and Finland during the northern hemisphere influenza season 2015/16

**DOI:** 10.2807/1560-7917.ES.2017.22.18.30527

**Published:** 2017-05-04

**Authors:** Hélène Bricout, Anne Laure Chabanon, Audrey Souverain, Christine Sadorge, Timo Vesikari, Timothy David Caroe

**Affiliations:** 1 Sanofi Pasteur MSD, Lyon, France; 2 Vaccine Research Center, University of Tampere, School of Medicine, Tampere, Finland; 3 Lighthouse Medical Practice, Eastbourne, East Sussex, United Kingdom

**Keywords:** Influenza, Influenza virus, Surveillance, vaccine, immunisation, vaccine safety, routine pharmacovigilance

## Abstract

Enhanced safety surveillance (ESS) was conducted in the United Kingdom and Finland for Vaxigrip and Intanza 15 µg to comply with the European Medicines Agency interim guidance aimed to detect any potential increase in reactogenicity in near real time following the annual update of the influenza vaccine strain composition. This pilot passive ESS was established to strengthen safety monitoring by facilitating spontaneous vaccinee reports and estimating near real-time vaccinee exposure. The primary objective was to estimate the reporting rates of suspected adverse reactions (ARs) occurring within 7 days post vaccination during the northern hemisphere 2015/16 influenza season. Among the Vaxigrip vaccinees (n = 1,012), 32 (3.2%) reported a total of 122 suspected ARs, including 110 suspected ARs that occurred within 7 days post vaccination. Among the Intanza 15 µg vaccinees (n = 1,017), 31 (3.0%) reported a total of 114 suspected ARs, including 99 that occurred within 7 days post-vaccination. These results were consistent with the known safety profile of the two vaccines and did not show any change in reactogenicity or safety concerns. This passive ESS showed improved data reporting and demonstrated its suitability to health authorities’ requirements; further fine tuning of the methodology is under discussion between all stakeholders.

## Introduction

Influenza is an acute viral respiratory infection that affects 5% to 20% of the global population annually [1]. This rate amounts to ca 25 to 100 million persons each influenza season in Europe. The epidemiology of seasonal influenza has been well characterised, particularly in the northern hemisphere (NH), where the influenza season typically falls between November and April [2].

Vaccination is the only preventive measure for seasonal influenza. As recommended by the World Health Organization (WHO), the current trivalent or quadrivalent marketed influenza vaccines are composed of antigens from two influenza A strains and one or two influenza B virus strain [1]. The recommendation is based on extensive surveillance of influenza strains through the WHO Global Influenza Surveillance network as the influenza strains continue to evolve, causing an antigenic mismatch between the virus strains in the vaccine and the circulating viruses in the subsequent influenza season [3,4]. Consequently, the strain composition of the influenza vaccine is adapted to the epidemiological situation to provide optimal protection for the population.

The European Medicines Agency (EMA) requests annual enhanced safety surveillance (ESS) for all seasonal influenza vaccines. The purpose of this requirement is to rapidly detect a clinically significant change (beyond what was known or expected with the previous vaccine composition) in the frequency and/or severity of reactogenicity (local, systemic or allergic reactions) that may indicate the potential for more serious risks as exposure to the vaccine increases. To avoid false attribution of such a signal to the general intrinsic safety profile of a product, it is recommended that ESS should involve subanalysis of more than one batch [5].

Interim guidance was issued by the Pharmacovigilance Risk Assessment Committee (PRAC) in April 2014 to outline the principles to be followed for improved continuous routine surveillance of influenza vaccines [5]. Experiences and limitations faced during the NH 2014/15 pilot influenza season were discussed between the vaccine marketing authorisation holders (MAHs) through a dedicated safety task force within Vaccines Europe (European Vaccines Manufacturers Association within the European Federation of Pharmaceutical Industries and Associations (EFPIA)) and were presented to the EMA/PRAC/Vaccine Working Party in November 2014. By December 2014, the PRAC recommended establishing a passive ESS for the NH 2015/16 influenza season to the MAHs. Thus, a new design was developed to monitor Vaxigrip (intramuscular trivalent split-virion inactivated influenza vaccine) and Intanza 15 µg (intradermal trivalent split-virion inactivated influenza vaccine) reactogenicity that relied on enhanced routine pharmacovigilance early in the influenza season.

In the United Kingdom (UK), Vaxigrip is recommended for adults older than 65 years, risk groups between 18 and 64 years and children between 6 months and 2 years of age. In Finland, Vaxigrip is recommended to be used in children 6 months to 2 years of age and in at-risk groups from 3 to 18 years of age. Children aged 2 to 3 years in Finland and 2 years and older in the UK preferentially receive another influenza vaccine (live attenuated influenza vaccine) per respective national recommendations [6,7]. Intanza 15 µg is only used in the UK and recommended for individuals 60 years and older. Notably, the Vaxigrip trade name in the UK is Inactivated Influenza (split virion) BP vaccine, but it will be referred to as Vaxigrip in this manuscript.

The principle of this passive ESS was to rapidly estimate vaccine usage or coverage (number of vaccinees or doses administered) and to facilitate passive reporting of suspected adverse reactions (ARs) from vaccinees in order to derive AR reporting rates from the same source of population. For these spontaneous reports, causality assessment was not requested from the vaccinee or healthcare professionals (HCPs) and was not performed by the MAH.

The primary objective was to estimate the reporting rates of suspected ARs occurring within 7 days following routine vaccination with Vaxigrip or Intanza 15 µg during the NH 2015/16 influenza season. The secondary objectives were to estimate the reporting rates of suspected ARs occurring within 7 days following routine vaccination with Vaxigrip or Intanza 15 µg according to age group and of serious suspected ARs post vaccination not limited to 7 days. This ESS also aimed to provide reference reporting rates for comparison in the next influenza season (2016/17). As an exploratory objective, a batch analysis would be performed if a signal was detected, whenever possible, to avoid false attribution of the signal to the general intrinsic safety profile of the product.

## Methods

### Design

This was a multicentre, non-interventional, observational, passive ESS conducted in the UK and Finland to ensure the representativeness of all age groups indicated for each vaccine and the use of at least two different batches. The passive ESS relied on enhanced (facilitated) reporting of suspected ARs by increasing the awareness of vaccinees, through trained HCPs, regarding the importance of reporting suspected ARs post vaccination (especially those occurring within 7 days post vaccination) and by distributing safety report cards (SRCs) that allowed vaccinees to report suspected ARs through a dedicated toll-free telephone number. Near real-time, age-specific, brand-specific influenza vaccination coverage was achieved in addition to near real-time analysis estimating suspected AR reporting rates within 7 days post vaccination during the NH 2015/16 influenza season.

### Setting

The passive ESS started on 13 October 2015 for Vaxigrip and 17 October 2015 for Intanza 15 µg and ended when 1,000 SRCs each had been distributed (on 2 December 2015 for Vaxigrip and on 8 December 2015 for Intanza 15 µg). Any reports received outside the ESS period were handled as routine spontaneous reports but were not included in the analysis.

### Participants

Vaccinees who received Vaxigrip or Intanza 15 µg in routine practice during the NH 2015/16 influenza season and who accepted the SRC (or their parents, in cases of child vaccinees) were eligible for participation in this ESS. There were no exclusion criteria.

### Procedures and data collection method

A paper SRC specific to Vaxigrip or Intanza 15 µg provided the following information to the vaccinee: details regarding the ESS, instructions on how to report suspected ARs, the dedicated local toll-free telephone number, the site identifier, a unique SRC identification number, vaccine brand and batch, vaccination date and name of the treating physician.

Vaccine coverage data were collected at practice level by the HCP/vaccinator(s) on a real-time basis (at least once a day) using an electronic data capture system. Vaccinees were encouraged to report any suspected post-vaccination ARs, especially those occurring within 7 days (although reports of ARs after 7 days were also considered for the analysis). A structured telephone interview was developed to ensure the appropriateness and completeness of data collection when vaccinees called to report suspected ARs.

All events reported spontaneously by vaccinees were considered suspected ARs and were recorded and reported according to Good Pharmacovigilance Practice module VI [8]. All suspected ARs were described. PRAC Adverse Events of Interest (AEIs), as listed in the guidance, were also specifically described [5]. Per protocol, side effects reported by a vaccinee or HCP were considered suspected ARs (unless the reporters specifically stated the events to be unrelated or excluded a causal relationship).

Safety signals were defined per Good Pharmacovigilance Practice Annex I revision 3 [9].

### Population size

The number of SRCs needed to be distributed per brand (n = 1,000) was estimated based on the expected AR reporting rate and the ability to detect common or very common ARs. The number of sites (six in Finland and 14 in the UK) was based on the expected volume of vaccinations with Vaxigrip and Intanza 15 µg and their ability to distribute SRCs within a short time period. Age representativeness of the population was ensured through country/site selection (in Finland, only paediatric vaccination centres were selected); nevertheless, the SRC distribution at site level followed routine vaccination practices. The number of vaccinees who would potentially report suspected ARs could only be stimulated but not controlled.

### Statistical analysis

The ESS population included all vaccinees who were vaccinated in routine practice with either Vaxigrip or Intanza 15 µg and who received the SRC. No confirmatory hypothesis testing was conducted for the analyses. All analyses were descriptive and were produced using SAS version 9.2. Verbatim ARs were coded with Medical Dictionary for Regulatory Activities terminology (version 18.0) and processed according to routine pharmacovigilance processes.

ESS reporting rates were calculated per brand using the following formula:

ESS reporting rate = (Number of vaccinees reporting ARs within 7 days x 100) / total number of SRCs distributed

Suspected AR reporting rates were estimated per brand using the following method:

Suspected AR reporting rate = (Number of ARs within 7 days x 100) / total number of SRCs distributed

Confidence intervals (CIs) for ESS reporting rates were computed using the Wald method if the AR count was ≥ 5 and using exact method if the AR count was < 5.

All suspected ARs (including PRAC AEIs, serious suspected ARs and other suspected ARs) and corresponding AR reporting rates were reported and summarised by vaccine, age groups (Vaxigrip: 6 months to < 6 years; ≥ 6 years to < 13 years; ≥ 13 years to < 18 years, ≥ 18 years to ≤ 65 years, and > 65 years; Intanza 15 µg: ≥ 60 years), seriousness (Yes/No), severity (Grade 1 (mild), Grade 2 (moderate), Grade 3 (severe), and unknown per protocol severity definition), and day of onset since vaccination (≤ 7 and > 7 days). A similar analysis was also performed on serious suspected ARs.

The mean number of ARs per vaccinee who reported at least one suspected AR was also calculated. For each brand, weekly reports for signal detection were generated and analysed. A 1-month interim report (1 month after the first SRCs were distributed) and a final report were compiled and submitted to the relevant health authorities. AR reporting rates were calculated and compared with the frequency of the AEIs reported during the NH 2014/15 influenza season clinical trials and with the expected rates based on current product-specific data from the Summary of Product Characteristics (SmPC) [10]. No statistical tests were performed [11].

### Ethics

The ESS was conducted in accordance with Good Epidemiological Practice, the European Network of Centres for Pharmacoepidemiology and Pharmacovigilance Guide on Methodological Standards in Pharmacoepidemiology [12,13] and Good Pharmacovigilance Practices [8]. The ESS was submitted to national authorities as required by the local regulations and was approved by national ethics committees.

## Results

### Exposure data

A total of 1,012 SRCs for Vaxigrip and 1,017 SRCs for Intanza 15 µg were distributed to different age groups in the UK and Finland during the 8-week period from 13 October to 8 December 2015 (Table 1). We also considered in the analysis additional SRCs distributed on the same day the 1,000th SRC was reached. 

**Table 1:** Safety report cards distributed for Vaxigrip and Intanza 15 µg vaccinees, by age group, United Kingdom and Finland, 2015/16 (n = 2,029)

Age group	Safety report cards distributed	
	Number	Percentage
**Vaxigrip**		
6 months to < 6 years	496	49.0
≥ 6 to < 13 years	111	11.0
≥ 13 to < 18 years	19	1.9
≥ 18 to ≤ 65 years	149	14.7
> 65 years	237	23.4
Total Vaxigrip	1,012	100.0
**Intanza^a^**		
Total Intanza	1,017	100.0

^a^ All Intanza 15 µg vaccinees were ≥ 60 years-old.

The ESS covered 21 different batches of Vaxigrip and three different batches of Intanza 15 µg. Approximately half (51%) of the Vaxigrip vaccinees received the same batch; the other half (49%) received Vaxigrip from 20 different batches. Almost all of the Intanza vaccinees (except three vaccinees) received the same batch. Because no safety signal was detected for either Vaxigrip or Intanza 15 µg, no specific batch analysis was conducted.

### Vaxigrip safety data

Among the Vaxigrip vaccinees, 32 (3.2%) reported a total of 122 suspected ARs (mean of 3.8 ARs/vaccinee who reported at least one AR), including 110 suspected ARs that occurred within 7 days post-vaccination (Table 2).

**Table 2:** Summary of suspected adverse reactions by age group, time of onset and brand, United Kingdom and Finland, 2015/16 (n = 2,029)

	Time of onset after vaccination								
	≤ 7 days			> 7 days		Total^a^			
	n		%	n	%	n	%		
**Vaxigrip (n = 1,012)**									
Total number of suspected AR	110	10.9		12	1.2	122		12.1	
Total number of PRAC AEI	42	4.1		4	0.4	46		4.5	
Total number of vaccinees with at least 1 suspected AR	31	3.1		3	0.3	32		3.2	
Total number of vaccinees with PRAC AEI	22	2.2		3	0.3	25		2.5	
**6 months to < 6 years (n = 496)**									
Number of suspected AR	40	8.1		2	0.4	42		8.5	
Number of PRAC AEI	20	4.0		1	0.2	21		4.2	
Number of vaccinees with at least 1 suspected AR	14	2.8		1	0.2	14		2.8	
Number of vaccinees with PRAC AEI	11	2.2		1	0.2	12		2.4	
**≥ 6 to < 13 years (n = 111)**									
Number of suspected AR	8	7.2		0	0	8		7.2	
Number of PRAC AEI	7	6.3		0	0	7		6.3	
Number of vaccinees with at least 1 suspected AR	2	1.8		0	0	2		1.8	
Number of vaccinees with PRAC AEI	2	1.8		0	0	2		1.8	
**≥ 13 to < 18 years (n = 19)**									
	No data reported for this age group								
**≥ 18 to ≤ 65 years (n = 149)**									
Number of suspected AR	12	8.0		0	0	12			8.0
Number of PRAC AEI	4	2.7		0	0	4			2.7
Number of vaccinees with at least 1 suspected AR	4	2.7		0	0	4	2.7		
Number of vaccinees with PRAC AEI	2	1.3		0	0	2	1.3		
> **65 years (n = 237)**									
Number of suspected AR	50	21.1		10	4.2	60		25.3	
Number of PRAC AEI	11	4.6		3	1.3	14		5.9	
Number of vaccinees with at least 1 suspected AR	11	4.6		2	0.8	12		5.1	
Number of vaccinees with PRAC AEI	7	3.0		2	0.8	9		3.8	
**Intanza^b^ (n = 1,017)**									
Total number of suspected AR	99	9.7		15	1.5	114		11.2	
Total number of PRAC AEI	53	5.2		3	0.3	56		5.5	
Total number of vaccinees with at least 1 suspected AR	29	2.9		3	0.3	31		3.0	
Total number of vaccinees with PRAC AEI	26	2.6		3	0.3	28		2.8	

AEI: adverse event of interest; AR: adverse reaction; PRAC: pharmacovigilance risk assessment committee.

^a^ Not all numbers add up as vaccinees could report suspected AR in both time intervals.

^b^ All Intanza 15 µg vaccinees were ≥ 60 years-old.

The highest reporting rate of suspected ARs occurring within 7 days post vaccination was observed in vaccinees older than 65 years; 11 of these vaccinees reported 50 suspected ARs (4.5 ARs within 7 days/vaccinee who reported at least one AR; Table 2).

There was no obvious distribution pattern in the type of suspected ARs across age groups, with the majority of individual ARs occurring at a frequency of less than 1%. The total number of suspected ARs that occurred at a frequency of 1% or higher are presented by age group and time of onset in Table 3.

**Table 3:** Most frequently reported suspected adverse reactions (with reporting rates  **≥** 1% ) by age group and time of onset, United Kingdom and Finland, 2015/16 (n = 2,029)

Preferred term	Time of onset									
	≤ 7 days				> 7 days			Total		
	n	%		CI	n	%	CI	n	%	CI
**Vaxigrip (n = 1,012)**										
**6 months to < 6 years (n = 496)**										
Cough	5	1.0		0.1–1.9	0			5	1.0	0.1–1.9
Pyrexia	7	1.4		0.4–2.4	1	0.2	0.0–1.1	8	1.6	0.5–2.7
Rhinorrhoea	5	1.0		0.1–1.9	1	0.2	0.0–1.1	6	1.2	0.2–2.2
**≥ 6 to < 13 years (n = 111)**										
Vaccination site erythema	2	1.8		0.2–6.4	0			2	1.8	0.2–6.4
**≥ 13 to < 18 years (n = 19)**										
	No data reported for this group									
**≥ 18 to ≤ 65 years (n = 149)**										
	No suspected AR ≥ 1% of total reported for this group									
**> 65 years (n = 237)**										
Cough	3	1.3		0.3–3.7	1	0.4	0.0–2.3	4	1.7	0.5–4.3
Fatigue	2	0.8		0.1–3.0	1	0.4	0.0–2.3	3	1.3	0.3–3.7
Headache	3	1.3		0.3–3.7	1	0.4	0.0–2.3	4	1.7	0.5–4.3
Influenza-like illness	5	2.1		0.3–3.9	0			5	2.1	0.3–3.9
Malaise	3	1.3		0.3–3.7	2	0.8	0.1–3.0	5	2.1	0.3–3.9
Nasopharyngitis	3	1.3		0.3–3.7	0			3	1.3	0.3–3.7
Oropharyngeal pain	2	0.8		0.1–3.0	1	0.4	0.0–2.3	3	1.3	0.3-3.7
**Intanza** ^b^ **15 µg (n = 1,017)**										
Vaccination site pain	10		1.0	0.4–1.6	0			10	1.0	0.4-1.6

AR: adverse reaction; CI: confidence interval.

^a^ Not all numbers add up as vaccinees could report suspected AR in both time intervals.

^b^ All Intanza 15 µg vaccinees were ≥ 60 years-old.

One serious suspected AR was reported following Vaxigrip vaccination. A person in their late 70s experienced a chest infection (lower respiratory tract infection, which was considered to be an important medical event) 18 days after vaccination, which started with sore throat, headache, coughing and feeling ‘unpleasant’ and hot. The vaccinee's medical history included a previous chest infection 2 weeks before the influenza vaccination. The vaccinee was later reported to be recovering from the second chest infection following vaccination.

Overall, 46 suspected PRAC AEIs were reported by 25 vaccinees (1.8 suspected AEIs/vaccinee who reported at least one AR). Of these AEIs, 42 suspected AEIs occurred within 7 days post vaccination (Table 2). The most frequent (n ≥ 2) PRAC AEIs with an onset within 7 days post vaccination are presented by severity in Table 4.

**Table 4:** Most frequently reported PRAC adverse events of interest (events reported at least twice) with onset within 7 days, by severity, United Kingdom and Finland, 2015/16 (n = 2,029)

Preferred term	Mild			Moderate			Severe			Unknown			Total		
	n	%	95% CI	n	%	95% CI	n	%	95% CI	n	%	95% CI	n	%	95% CI
**Vaxigrip (n = 1,012)**															
**Number of vaccinees with PRAC AEI**	9	0.9	0.3–1.5	3	0.3	0.1–0.9	5	0.5	0.1–0.9	9	0.9	0.3–1.5	22	2.2	1.3–3.1
Headache	0	0	0	0	0	0	1	0.1	0.0–0.5	4	0.4	0.1–1.0	5	0.5	0.1–0.9
Pyrexia	3	0.3	0.1–0.9	1	0.1	0.0–0.5	2	0.2	0.0–0.7	3	0.3	0.1–0.9	9	0.9	0.3–1.5
Vaccination site erythema	1	0.1	0.0–0.5	1	0.1	0.0–0.5	2	0.2	0.0–0.7	1	0.1	0.0–0.5	5	0.5	0.1–0.9
**Intanza 15 µg (n = 1,017)**															
**Number of vaccinees with PRAC AEI**	18	1.8	1.0–2.6	2	0.2	0.0–0.7	3	0.3	0.1–0.9	7	0.7	0.2–1.2	26	2.6	1.6–3.5
Malaise	3	0.3	0.1–0.9	0	0	0	1	0.1	0.0–0.5	2	0.2	0.0–0.7	6	0.6	0.1–1.1
Vaccination site erythema	6	0.6	0.1–1.1	1	0.1	0.0–0.5	0	0	0	2	0.2	0.0–0.7	9	0.9	0.3–1.5
Vaccination site pain	9	0.9	0.3–1.5	0	0	0	0	0	0	1	0.1	0.0–0.5	10	1.0	0.4–1.6
Vaccination site pruritus	4	0.4	0.1–1.0	0	0	0	1	0.1	0.0–0.5	0	0	0	5	0.5	0.1–0.9
Vaccination site swelling	5	0.5	0.1–0.9	0	0	0	0	0	0	0	0	0	5	0.5	0.1–0.9

AEI: adverse event of interest; PRAC: pharmacovigilance risk assessment committee.

Note: PRAC AEIs as listed in the guidance were specifically described as follows: Injection site reactions (pain, erythema, pruritus, swelling, induration and ecchymosis) and systemic reactions (fever > 38 °C, headache, malaise, myalgia, shivering, rash, vomiting, nausea, arthralgia, decreased appetite, irritability (for vaccinees younger than 5 years), crying (for vaccinees younger than 5 years), and events indicative of allergic and hypersensitivity reactions including ocular symptoms).

There was no obvious distribution pattern in the type of AEIs, their severity or their frequency observed across age groups for Vaxigrip. All AEIs were considered not serious.

### Intanza safety data

Among the Intanza 15 µg vaccinees, 31 (3.0%) reported 114 suspected ARs (3.7 ARs/vaccinee who reported at least one AR), including 99 suspected ARs that occurred within 7 days post vaccination (Table 2).

All of the suspected ARs were non-serious. One vaccinee could not be included in the analysis because of insufficient information to identify the SRC number. This vaccinee had reported the non-serious suspected ARs of cough and pain. The most frequently reported suspected ARs within 7 days post vaccination (those reported by ≥ 1% of vaccinees) are listed in Table 3.

Overall, 56 suspected PRAC AEIs were reported by 28 vaccinees (2 AEIs/vaccinee who reported at least one AR). Of these AEIs, 53 AEIs occurred within 7 days post vaccination (Table 2). All AEIs were considered non-serious. The most frequent (n ≥ 2) PRAC AEIs with an onset within 7 days post-vaccination are presented by severity in Table 4.

### Comparison of the reported frequencies with the reference data from the northern hemisphere 2014/15 enhanced safety surveillance

No increase was noted in the observed AEI frequencies for Vaxigrip or Intanza 15 μg during the NH 2015/16 ESS when compared with the frequencies observed during the NH 2014/15 ESS (data not shown).

### Comparison of the reported frequencies with the Summary of Product Characteristics

#### Vaxigrip

Influenza-like illness (ILI) was found to have a higher reporting frequency in this ESS compared with the Vaxigrip SmPC. ILI was reported by five vaccinees (2.1%; 95% CI: 0.3–3.9%) older than 65 years but was not reported by vaccinees aged 18–65 years, which led to a combined ILI reporting rate of 1.3%. This observed frequency was slightly higher than the ‘uncommon’ (≥ 0.1% to < 1%) frequency for ILI in the groups of adults and elderly people in the SmPC. However, the slightly higher reporting rate observed was not considered clinically relevant upon medical review. The other suspected ARs had frequencies lower than or equal to the SmPC frequencies (Table 5).

**Table 5:** Comparison of other reactions (not solicited in the northern hemisphere 2014/15 clinical trial) with the Vaxigrip Summary of Product Characteristics, United Kingdom and Finland, 2015/16 (n = 2,029)

Adverse reaction^a^	ESS 2015/16 (≤ 7 days)		Vaxigrip SmPC (≤ 7 days)	Frequency^b^		Comparison result
	Age group	Observed frequency per age group	Age group	SmPC	ESS 2015/16	Higher or equal or lower than SmPC
Diarrhoea	6 months to < 6 years	0.2%	6 to 35 months	Very common	Uncommon	Lower
Diarrhoea	≥ 18 years^c^	0.3%	≥ 18 years	Uncommon	Uncommon	Equal
Dizziness	≥ 18 years^c^	0.5%	≥ 18 years	Uncommon	Uncommon	Equal
Influenza- like illness	≥ 18 years^c^	1.3%	≥ 18 years	Uncommon	Common	Higher
Asthenia	≥ 18 years^c^	0.3%	≥ 18 years	Very common	Uncommon	Lower
Sweating increased	≥ 18 years^c^	0.3%	≥ 18 years	Common	Uncommon	Lower

ESS: enhanced safety surveillance; SmPC: summary of product characteristics.

Note: A vaccinee with multiple occurrences of an adverse reaction is counted only once under the applicable system organ class/preferred term.

^a^ Only not solicited adverse reactions in the northern hemisphere 2014/15 clinical trial and reported in this ESS are compared with the SmPC and included in this table.

^b^ Very common (≥ 1/10 or ≥ 10%); common (≥ 1/100 to < 1/10 or ≥ 1% to < 10%); uncommon (≥ 1/1,000 to < 1/100 or ≥ 0.1% to < 1%); rare (≥ 1/10,000 to < 1/1,000 or ≥ 0.01% to < 0.1%); very rare (< 1/10,000 or < 0.01%).

^c^ Combined age groups of adult and elderly vaccinees.

#### Intanza 15 µg

Fatigue and sweating (hyperhydrosis) were reported following Intanza vaccination, and the reported frequencies in the ESS were similar to those referenced in the SmPC (Table 6).

**Table 6:** Comparison of other reactions (not solicited in the northern hemisphere 2014/15 clinical trial) with Intanza 15 µg summary of product characteristics, United Kingdom and Finland, 2015/16 (n = 2,029)

Adverse reaction	ESS 2015/16 (≤ 7 days + > 7 days)		Intanza 15 µg SmPC (≤ 7 days + > 7 days)	Frequency^b^		Comparison result
	Age group^c^	Reported frequency per age group	Age group	SmPC	ESS 2015/16	Higher or equal or lower than SmPC
Fatigue	≥ 18 years	0.2%	> 60 years	Uncommon	Uncommon	Equal
Sweating	≥ 18 years	0.2%	> 60 years	Uncommon	Uncommon	Equal

ESS: enhanced safety surveillance; SmPC: summary of product characteristics.

Note: A vaccinee with multiple occurrences of an adverse reaction is counted only once under the applicable system organ class/preferred term.

^a^ Only not solicited adverse reactions in the northern hemisphere 2014/15 clinical trial and reported in this ESS are compared with the SmPC and included in this table.

^b^ Very common (≥ 1/10 or ≥ 10%); common (≥ 1/100 to < 1/10 or ≥ 1% to < 10%); uncommon (≥ 1/1,000 to < 1/100 or ≥ 0.1% to < 1%); rare (≥ 1/10,000 to < 1/1,000 or ≥ 0.01% to < 0.1%); very rare (< 1/10,000 or < 0.01%).

^c^ Combined age groups of adult and elderly vaccinees.

## Discussion

In this ESS, vaccinees were encouraged to report any suspected ARs that they experienced, with an emphasis on those occurring within 7 days post vaccination. Hence, the reporting of suspected ARs was stimulated but remained spontaneous in nature (i.e. not solicited). We observed higher reporting rates when spontaneous notification was stimulated (3.2% for Vaxigrip and 3.0% for Intanza 15 µg) compared with reporting rates in routine pharmacovigilance (passive spontaneous non-stimulated system). Spontaneous reporting rates after seasonal influenza vaccination range from 20 to 90 reports per 1,000,000 people vaccinated [14-19]. Passive ESS has been shown to increase reporting rates two- to fivefold when switched from routine pharmacovigilance [20,21].

This study was executed in a time-efficient manner. Approximately 1.5–2 hours per HCP were dedicated to protocol training, processes to be used, management of vaccinees, site management and the end of the ESS process, depending on staff involved. The contact centre needed ca 15 min per vaccinee to record the suspected AR. However, information on the time spent per vaccinee by the HCP to explain the ESS, distribute the SRCs, and explain how ARs were to be reported was not collected as part of this ESS.

The strengths of this ESS were that the number of SRCs distributed was consistent with the estimated sample size and that weekly analyses were performed, which allowed for near real-time investigation of the reactogenicity of Vaxigrip and Intanza 15 µg. The safety reports received were well documented in terms of exposure data (brand, batch and date of vaccination), which is not always the case with routine pharmacovigilance. The overall reporting rates for the two products were of the same order of magnitude. By considering two countries and using a thorough site selection process, we were able to gather data across all age groups as recommended by the guidance, including data in paediatric age groups, over-represented compared with paediatric routine coverage rate.

Overall, the mean numbers of suspected ARs per vaccinee who reported at least one AR within 7 days post-vaccination were 3.8 for Vaxigrip and 3.7 for Intanza 15 µg, ranging between 0 (for vaccinees in the 13–18 years age group owing to the small number of SRCs distributed) and 13 (for vaccinees older than 65 years). The higher average number of suspected ARs in the group of elderly people could be due to the well-known correlation between increasing age and AR reporting rate. Frailty, medical history and concomitant use of medication are common causes of this phenomenon [22]. No obvious distribution pattern in the type and frequency of suspected ARs was observed across age groups for either vaccine.

All of the reported ARs were non-serious, except for one serious AR reported after Vaxigrip vaccination. The passive ESS results do not raise any concerns about the safety of Vaxigrip and Intanza 15 µg. None of the observed frequencies of AEIs in the current ESS were above the frequencies observed during the NH 2014/15 clinical study [11]. No safety issues were observed, and the safety profile of the two vaccines was consistent with what is known for both products. Per EMA interim guidance, data was to be generated from at least two batches of the vaccines. This requirement was fulfilled for Vaxigrip but was not feasible for Intanza 15 µg owing to the fragmented market share.

The passive ESS had the following potential limitations: firstly, there was no control over the actual reporting (under-reporting was still possible) or the timing of a suspected AR report relative to the time since vaccination (suspected ARs that occurred within 7 days could still be reported outside the ESS period). Secondly, the age groups in which the vaccine was used could not be controlled and depended on national recommendations for influenza vaccination, as well as the vaccine coverage rates per age group observed in routine practice. In addition, the choice to conduct the ESS in two countries, with Finland dedicated to the distribution of paediatric SRCs, affected the age group distribution for the SRC. During the ESS, all age groups were represented. However, for Vaxigrip, most of the SRCs were distributed in the age groups 6 months to < 6 years (n = 496) and in the age group older than 65 years (n = 237). Only 19 SRCs were distributed in the age group 13 to 18 years. Therefore, data from this specific paediatric age group were difficult to capture owing to low influenza vaccine coverage. Thirdly, some degree of selection bias may have occurred because vaccinees who accepted the SRC might have reported more (or fewer) ARs than those who refused the SRC. Moreover, HCPs could have preselected the vaccinees to whom the SRC was proposed, even if the instructions were to distribute the SRC on an ongoing basis to all eligible vaccinees. In addition, the vaccinees who received the vaccine early in the season might have been different from those who received the vaccine later in the season. However, this bias is most probably limited, as some sites distributed the SRCs very quickly to large vaccinee groups on days of massive organised influenza vaccinations.

Finally, some operational constraints were faced before initiating this ESS at the site level. In the UK, there was no official start date and influenza vaccination started by the middle of October in the context of ESS at the selected sites. In Finland, the national official start date for the influenza vaccination was 9 November 2015. The start date at the site level for the ESS depended on HCP availability for initiation, contract signature, local practice organisation for seasonal influenza vaccination and different local approval dates. The first site in Finland started SRC distribution on 10 November 2015, immediately after the official national start date for influenza vaccination. The ESS started as closely as possible to the time of the first influenza vaccinations in the selected sites in both countries; however, these start dates may have been some weeks after the first administration of Vaxigrip doses elsewhere in Europe and may have affected the speed at which potential safety issues could have been detected.

The estimated AR reporting rates will provide baseline AR reporting rates to improve comparison during the next NH influenza season (2016/17) using a similar passive methodology. A limitation in the current comparison is that the reference data from ESS NH 2014/15 were obtained from active safety surveillance (clinical trial) and not from spontaneous reporting. The finalisation of the guidance related to ESS is currently under discussion at EMA, and current passive ESS pilot experiences will provide data to further support recommendations. Despite the historic success of immunisation in reducing the morbidity and mortality of several diseases, some public concerns about the safety of vaccines remain. These concerns occasionally erode public confidence in immunisation and sometimes lead to vaccine hesitancy and disease outbreaks. Therefore, enhanced influenza vaccine safety monitoring can contribute to increase public confidence in vaccine safety.

In the absence of a more systemic, centralised, pan-European safety surveillance system, we believe that the passive ESS experience presents a suitable model for enhanced passive surveillance of seasonal influenza vaccines.

## Conclusions

The current pilot ESS used a passive approach and showed higher AR reporting rates than previously shown for routine spontaneous reporting. There was no obvious distribution pattern in the type and frequency of suspected ARs for Vaxigrip or Intanza 15 µg. We did not observe any clinically significant changes compared with what is known or expected for either vaccine, nor any safety concerns during the current ESS period. The ESS results have improved data reporting and demonstrated its suitability to health authorities’ requirements; further fine tuning of the methodology is under discussion between all stakeholders. A continuous dialogue between the MAHs (through Vaccines Europe) and the European health authorities will help optimise and scale up the ESS system for future seasons.
